# Dual use of e-cigarettes with conventional tobacco is associated with increased sleep latency in cross-sectional Study

**DOI:** 10.1038/s41598-022-06445-8

**Published:** 2022-02-15

**Authors:** Ira Advani, Deepti Gunge, Shreyes Boddu, Sagar Mehta, Kenneth Park, Samantha Perera, Josephine Pham, Sedtavut Nilaad, Jarod Olay, Lauren Ma, Jorge Masso-Silva, Xiaoying Sun, Sonia Jain, Atul Malhotra, Laura E. Crotty Alexander

**Affiliations:** 1 Pulmonary Critical Care Section, Veterans Affairs (VA) San Diego Healthcare System, La Jolla, CA 92161 USA; 2 Division of Pulmonary, Critical Care and Sleep Medicine, Department of Medicine, University of California San Diego (UCSD), La Jolla, CA 92093 USA; 3 Biostatistics Research Center, Herbert Wertheim School of Public Health and Human Longevity Science, UCSD, La Jolla, CA 92093 USA; 4 University of California, San Diego (UCSD), 9500 Gilman Dr, MC 9111J, San Diego, CA 92093 USA; 5 Internal Medicine program, Oklahoma State University Medical Center, Tulsa, Oklahoma USA

**Keywords:** Translational research, Sleep

## Abstract

The health effects of e-cigarettes remain relatively unknown, including their impact on sleep quality. We previously showed in a pilot study that females who smoke both conventional tobacco and vape e-cigarettes (dual users) had decreased sleep quality (measurement of how well an individual is sleeping) and increased sleep latency (amount of time to fall asleep), suggesting an influence by gender. Cough is also known to adversely impact sleep quality and may be caused by inhalant use. As a result, we undertook this study to assess the impact of e-cigarette, conventional tobacco, and dual use on sleep quality, sleep latency, cough, and drug use. Participants (n = 1198) were recruited through online surveys posted to social media sites with a monetary incentive. Participants were grouped by inhalant use, with 8% e-cigarette users, 12% conventional tobacco users, 30% dual users, and 51% non-smokers/non-vapers. Dual use of e-cigarettes and conventional tobacco was associated with increased sleep latency relative to non-smokers/non-vapers by multivariable linear regression (mean difference of 4.08; 95% CI: 1.12 to 7.05, raw p = 0.007, adjusted p = 0.042); however, dual usage was not significantly associated with sleep quality relative to non-smokers/non-vapers (mean difference 0.22, 95%CI: (−0.36, 0.80), raw p = 0.452, adjust p = 0.542). Dual use was also associated with a higher reporting of cough (p = 0.038), as well as increased marijuana (p < 0.001) and cocaine (p < 0.001) usage. This study demonstrates that  dual use is associated with longer sleep latency, and suggests that the shared component of nicotine may be a driver. Because sleep broadly impacts multiple aspects of human health, defining the associations of e-cigarettes and vaping devices on sleep is critical to furthering our understanding of their influence on the body.

## Introduction

The popularity of electronic cigarettes (e-cigarettes) and vaping devices such as JUUL has spiked in the last few years, especially amongst adolescents (age 11 to 17) and young adults (ages 18 to 34)^1,2^. Despite major increases in e-cigarette usage, there is a lack of data on potential health effects caused by these devices^3–5^. The e-cigarette or vaping device use-associated lung injury (EVALI) epidemic that started in 2019 illuminated the dangers of inhaling chemicals contained within these vaping aerosols^6,7^. Moreover, since active agents like nicotine and marijuana have an immediate impact on activating pathways of the central nervous system (CNS), inhalation of aerosols from e-devices may impact sleep^8^. The aim of this study was to determine if inhalant use of e-cigarettes, conventional tobacco, or dual use of both products would impact sleep quality (a measurement of how well an individual is sleeping), sleep latency (time it takes for an individual to fall asleep), presence of cough, and use of drugs.

We previously completed a small pilot study (274 participants) which suggested that women who smoked both conventional tobacco products and vaped e-cigarettes (dual users) had a significant decrease in their sleep quality^9^. Since dual users may intake greater quantities of nicotine than sole users of conventional tobacco or e-cigarettes^10^ we hypothesized that nicotine may have been the driving factor in our 2019 findings of diminished sleep quality in women who were dual users^11^. Moreover, women are known to have different susceptibilities to tobacco in comparison to men^12^, such as greater nicotine withdrawal-related distress and a greater urge to smoke to relieve this stress, which can cause further sleep disruptions^13^. Women also have more profound nicotine-induced acid reflux^14^ which can lead to poorer sleep quality. Due to these factors, one of the aims of our study was to determine if sex may play a role in susceptibility to the known sleep-disrupting effects of nicotine^15,16^. Also, since e-cigarette users most commonly use e-cigarette devices in conjunction with conventional tobacco cigarettes, dual users are a key population to study^17^.

Use of e-cigarettes has also been associated with increased cough^18–20^. Currently, it is known that cough can lead to sleep disruption, particularly in tobacco users^21^. With this larger study, we sought to assess the prevalence of cough in different types of inhalant users across ages with a specific question regarding whether dual use leads to the highest rates of cough across inhalant users. We view cough as an important patient-reported outcome; however, the data generated from the study cannot define definitive causal pathways. Further, we sought to assess the impact of cough on sleep disruption.

Nicotine can serve as a gateway drug and create a priming effect for drugs such as cocaine and methamphetamines^22^. For example, some studies suggest that cigarette smoking may facilitate the start of stimulant addiction^23,24^, which may explain the higher rates of cigarette smoking amongst cocaine and methamphetamine users in comparison to the general population^25^. Because it is important to public health to understand the potential impact of new inhalants on illicit drug use, we undertook these studies to determine potential associations between use of tobacco or e-cigarettes with use of non-nicotine drugs, such as marijuana or cocaine^26,27^.

In conclusion, we conducted this social media platform-based, reward-incentivized, anonymous research survey to determine whether dual users have worsened sleep quality and cough. While other studies have revealed an association between e-cigarettes or conventional tobacco and sleep disturbances^11,16,28^, there is little research on how dual usage, which is an increasingly common pattern of nicotine usage^17^, can impact these health outcomes. As a result, the objective of this larger study (1198 participants) across age groups was to assess whether inhalant use patterns were associated with alterations in sleep quality, cough, and drug use.

## Methods

### Participants

A total of 1198 individuals participated in our cross-sectional study and completed the online survey. Recruitment of participants started on November 11th, 2019 and ended recruitment on March 5th, 2020. While more responses were received after March 5th, responses past this date were excluded as they may have been related more to the effects of the COVID-19 pandemic versus typical inhalant usage. Participants were recruited via online surveys through wide-spread Twitter, Facebook, Craigslist and Reddit advertisements. In order to avoid sampling bias, online advertisement titles were varied and also posted in varying state forums with about 2–3 cities per state.

Our target population of the study was inhalant users of all ages. Because the goal of the study was to assess for different measures of health outcomes, our study was designed to recruit inhalant users of all ages. The primary eligibility criteria was that participants must be 13 and above. Participants ages 13 and above, who complete the entirety of the survey with valid responses, were included in the analysis portion. Participants who were below the age of 13 or included inappropriate and/or invalid responses were excluded from the study. There were no other exclusion criteria besides age and invalid responses. All studies were carried out in accordance with national human subjects research guidelines, with all participants providing informed consent. All protocols were approved by the University of California, San Diego (UCSD) institutional review board (IRB protocol # 160204). Participants were incentivized to complete online surveys through random lottery. IP addresses were recorded to identify participant’s location as well as prevent multiple responses per device.

### Materials

Inhalant use patterns were assessed utilizing the UCSD Inhalant Survey, which is updated every 6–12 months to accurately assess use of new e-devices, inhalants and vaping methods^9,29^. Sleep quality was determined using the Pittsburgh Sleep Quality Index (PSQI)^11,18^, a validated self-reporting questionnaire that identifies the nature of possible sleep problems. Overall PSQI scores (0–21) are used to analyze sleep quality, and the self-assessment includes questions to identify sleep habits, such as sleep latency. Sleep latency was determined through the following open-ended question included within the Pittsburgh Sleep Quality Index, “During the past month, how long (in minutes) has it taken you to fall asleep each night?”. Sleep latency can be an important indicator of sleep quality. For example, individuals with insomnia tend to have higher sleep latencies and enter slow wave sleep at a slower rate in comparison to healthy sleepers^30^. Furthermore, prolonged sleep latency may also be indicative of autonomic dysfunction which can affect progress in falling asleep^31^. Finally, cough symptoms and severity were assessed with the Leicester Cough Questionnaire (LCQ), a validated self-reporting questionnaire that is used to identify the presence of cough, as well as cough symptoms and impact through physical, social, and psychological domains^11,18^. All three surveys plus demographic questions and those to quantify marijuana and other recreational drug use were combined into one online survey (Supplementary Materials, Appendix A). To maintain a relatively short questionnaire and thus high completion rate, questions about comorbidities that could have impacted sleep quality were not included. STROBE guidelines were utilized in the preparation of the manuscript^32^ (Supplemental Materials, Strobe Statement).

### Exposure, outcomes, and covariates

Participants were first asked demographic questions—including their age, demographic, race, and ethnicity (Appendix A). Next, participants were asked “Have you ever used any form of tobacco”; those who answered “yes” were asked a follow up question: “Are you actively using any form of tobacco (> 1 use per month in the last year). Participants who answered “yes” to the first question were classified as ever Conventional Tobacco Users. Afterwards, participants were asked “Have you ever used an e-cigarette?”; those who answered “yes” were asked a follow up question: “Are you actively using any e-devices (> 1 use per month in the last year). Participants who answered “yes” to the first question were classified as ever E-cigarette users. Participants who answered “yes” to using tobacco and “yes” to using e-cigarettes were classified as ever Dual Users. Finally, participants who answer “no” to using tobacco and “no” to using e-cigarettes were classified as Non-smokers/Non-vapers. The following include the classification of the four inhalant groups analyzed in the study—“Non-smokers/non-vapers, e-cigarette users, conventional tobacco users, and dual users”.

Since this study was a follow-up to our pilot study published in AJRCC, we had well-defined hypotheses that we based our study design on. We developed six study objectives prior to the launch, completion, and analysis of our study. Our primary objective was to determine whether there are differences in sleep quality (determined by PSQI scores) between inhalant use group. Our five secondary objectives included the following: Are there differences in sleep quality between genders across inhalant groups?; Are there differences in cough incidence and severity (determined by LCQ score) between inhalant groups?; Is increased sleep latency (the amount of time it takes to fall asleep) the driving factor behind poor sleep quality in inhalant users?; Is any individual component of the LCQ the primary driver of poor sleep quality?; Is there a higher usage of recreational drugs amongst inhalant users (e-cigarette, conventional, dual users) compared to non-inhalant users (non-smokers/non-vapers)?

We focused on these six study objectives, and these were the only analyses conducted for this study. We did not design additional questions after the primary analysis was complete; no study objectives were developed after the analysis of the data.

### Analysis

Based on the pilot study, with 274 participants, mean difference in PSQI between the two groups was 1.68. Assuming a standard deviation of 4, which is a larger estimate from the two groups, the study has 99% power with n = 210 in each group. As a result, our study is sufficiently powered with a total about 1000 participants.

Descriptive analyses were performed to summarize the data by inhalant use groups. Group comparisons used analysis of variance (ANOVA) tests for the continuous variables (age) and Chi-square tests for the categorical variables (gender, race, ethnicity). A multivariable linear regression model was performed to study the association of inhalant use group with sleep quality, adjusting for age and gender. Mean differences (and 95% CI) in PSQI scores were estimated for e-cigarette users vs. non-smokers, dual users vs. nonsmokers, dual users vs. e-cigarette only users, dual users vs. conventional cigarette users, and conventional cigarette users vs. non-smokers. We also assessed whether the association of inhalant groups on sleep quality differed between males and females by including an interaction term between gender and inhalant groups in the model. A similar analysis was conducted for the other continuous outcomes. A logistic regression model was performed to study the association of inhalant use group with reporting presence of cough in the past 30-days. All models were adjusted for the following potential confounding variables: age, gender, race, and ethnicity. OR (and 95% CI) were estimated for all pairwise inhalant group comparisons. P-values for pairwise group comparisons were adjusted using the method of Benjamini & Hochberg (1995)^33^. Statistical software R (version 3.6.1) was used for the analysis (http://www.r-project.org). A sensitivity analysis was conducted with the 3 countries that had the majority of respondents—United States (n = 687), United Kingdom (n = 138), and Canada (n = 26).

## Results

### Demographics of survey participants

We received responses to surveys from 1198 individuals, with a 77.9% completion rate; thus, 933 responses were complete data and were included in the study. Surveys were excluded for analysis due to incomplete or invalid responses–those with unrelated or uninterpretable responses to the question(s). In total, 195 incomplete responses and 69 invalid responses were received and thus removed from the study for analysis. Every survey response was reviewed individually and those that consisted of inappropriate answers (such as numeric inconsistencies) were deemed invalid.

**Figure 1:**
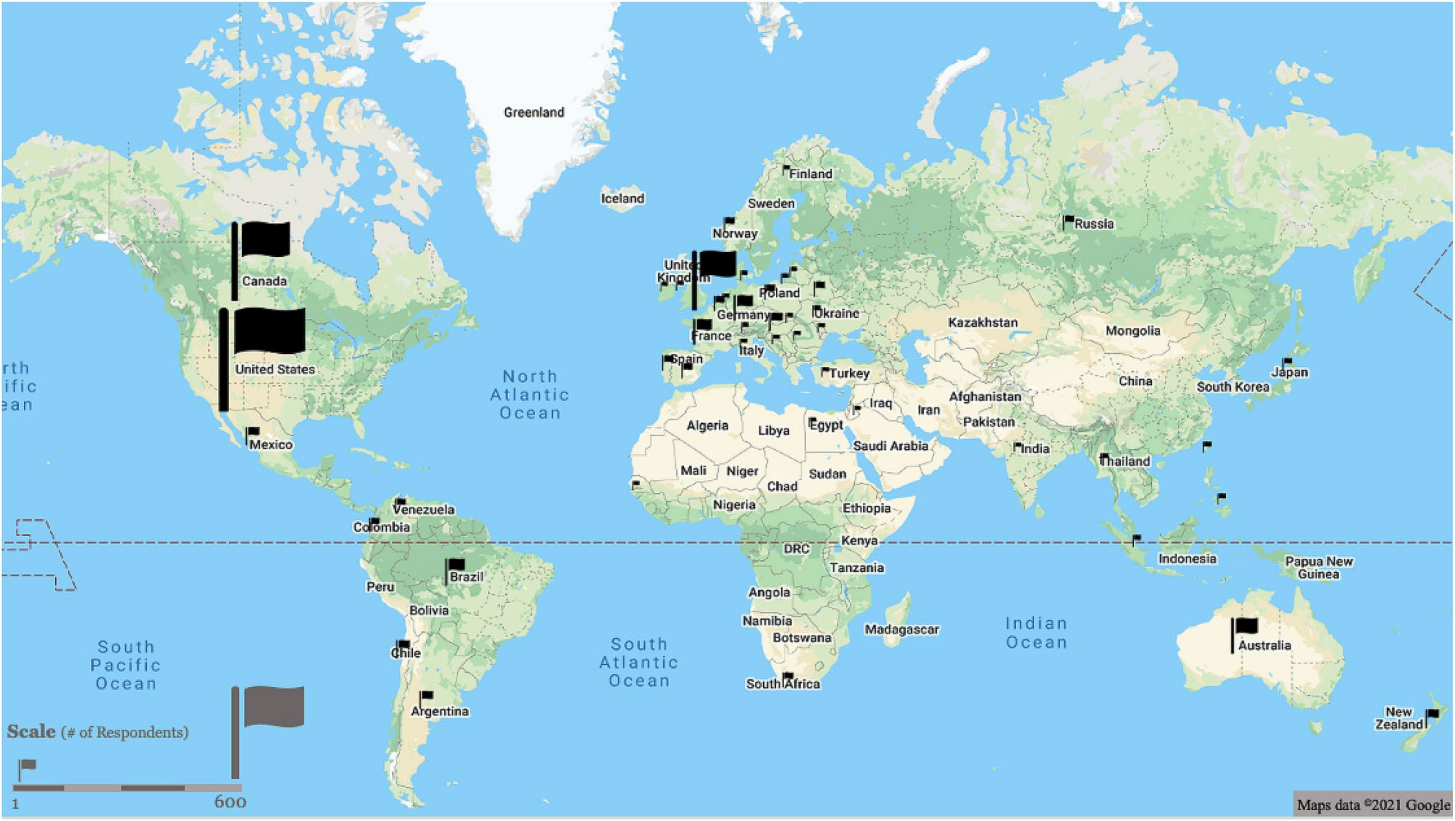
Responses were collected from 47 countries across 6 continents. Locations of the survey responders, by IP address. Responses were collected from over 47 countries and the size of each flag symbol on the map is proportional to the number of responses received from the region. For reference, the number of responses from the US was 687, from the United Kingdom was 138, from Brazil was 7, and from New Zealand was 2. Maps data ©2021 Google.

Mapping of survey response IP addresses demonstrated the international reach obtained by the social media advertisements utilized, with responses from 47 countries across 6 continents (Fig. 1). Reported ages of participants ranged from 13 to 70 years, with a mean of 26 years. Gender distribution was fairly even with 53% identifying as female, 46% as male, and 1% as non-binary or who refused to answer. The majority of respondents were Caucasian (62%), with the next highest percent being Asian (19%) and Asian Indian (6%; Table 1).

**Table 1:** Demographics of Survey Responders.

	Nonsmoker	Conventional	E-cig user	Dual user	Overall	p value
	n = 472	n = 109	n = 74	n = 278	n = 933	
**Age**						
Mean (SD)	22.6 (12.1)	34.6 (11.8)	18.4 (3.9)	30.6 (11.9)	26.1 (12.6)	< 0.001
**Gender**						
Female	265 (56.14%)	70 (64.22%)	49 (66.22%)	112(40.29%)	496 (53.16%)	< 0.001
Male	200(42.37%)	39 (35.78%)	24 (32.43%)	163 (58.63)	426 (45.66%)	
Other	7 (1.48%)	0 (0%)	1 (1.35%)	3 (1.08%)	11 (1.18%)	
Total	472 (99.99%)	109 (100%)	74 (100%)	278 (100%)	933 (100%)	
**Race**						
Alaska native	1 (0.21%)	2 (1.85%)	0 (0%)	0 (0%)	3 (0.32%)	0.001
Asian Indian	18 (3.84%)	9 (8.33%)	9 (12.16%)	17 (6.14%)	53 (5.71%)	
Asian	123 (26.23%)	8 (7.41%)	14 (18.92%)	27 (9.75%)	172 (18.53%)	
African American	7 (1.49%)	3 (2.78%)	0 (0%)	5 (1.81%)	15 (1.62%)	
Pacific Islander	4 (0.85%)	0 (0%)	1 (1.35%)	1 (0.36%)	6 (0.65%)	
White	260 (55.44%)	80 (74.07%)	37 (50%)	202 72.92%)	579 (62.39%)	
Middle Eastern	0 (0%)	0 (0%)	0 (0%)	2 (0.72%)	2 (0.22%)	
**Ethnicity**						
Hispanic	7 (1.49%)	0 (0%)	2 (2.7%)	4 (1.44%)	13 (1.4%)	< 0.009
Mixed	38 (8.1%)	6 (5.56%)	10 (13.51%)	15 (5.42%)	69 (7.44%)	
Prefer not to answer	11 (2.35%)	0 (0%)	1 (1.35%)	4 (1.44%)	16 (1.72%)	
Total	469 (100%)	108 (100%)	74 (99.99%)	277 (100%)	928 (100%)	

*Comparisons among the 4 groups are all significant with p < 0.001. ANOVA test was used for age; and Fisher exact test was used for gender and ethnicity.

The overall study sample was divided into four inhalant groups: non-smokers/non-vapers (51%), conventional tobacco smokers (12%), e-cigarette users (8%), and dual users of e-cigarettes and conventional tobacco (30%; Table 1). There were significant differences in age across inhalant groups (p < 0.001). Conventional tobacco users had the highest mean age (35 years, SD = 11.8), while e-cigarette users had the lowest mean age (18 years, SD = 3.9; Table 1). For dual users, the percentage of Caucasian respondents was highest at 73%, followed by Asian at 10%, and Asian Indian at 6%. E-cigarette users had a lower percentage of Caucasian respondents (50%) and higher mixed race at 14%. The racial ethnicity self-identification in the conventional tobacco user group was similar to dual users; however, non-smokers/non-vapers had a lower percentage of Caucasian respondents (55%) and a higher percentage of Asian respondents (26%). There was a low number of responses from the following: Middle Eastern (0.2%), Alaska Native (0.3%), Pacific Islander (0.7%), Hispanic (1.4%), African American (1.62%), and mixed ethnicities (7.4%).

### Dual use and female gender are associated with longer sleep latency

Sleep latency was associated with inhalant type, with dual users having longer sleep latency compared to non-smoking/non-vaping participants (mean difference of 4.08; 95% CI: 1.12 to 7.05, raw p = 0.007, adjusted p = 0.042) (Table 2). Gender was also independently associated with sleep latency, with males having shorter sleep latency compared to females (mean difference of -2.89, 95% CI: -5.38 to -0.40, p = 0.023), such that males in this study sample were found to fall asleep faster than females. There was no significant interaction between gender and inhalant groups on sleep latency (p = 0.487). However, both male and female dual users reported longer sleep latency relative to non-smoking/non-vaping participants (3.42 and 4.51 min, respectively). Finally, race was also associated with sleep latency as Asian participants had significantly shorter sleep latency compared to Caucasian participants (mean difference = −3.83, 95%CI (−6.93, −0.73), p = 0.016).

**Table 2:** Multivariable linear regression model to assess the association between inhalant groups and sleep latency by PSQI.

	Mean difference	95% CI	Wald Chi-squared	p-value
Age	0.04	(−0.07, 0.15)	0.42	0.52
**Gender**				0.023
Male vs female	−2.89	(−5.38, −0.40)	5.16	
**Race**			6.10	0.047
Asian vs. White	−3.83	(−6.93, −0.73)		
Other vs. White	−0.14	(−4.08, 3.81)		
**Ethnicity**			4.78	0.092
Hispanic vs. non-Hispanic	−4.35	(−8.62, −0.08)		
Unknown vs. non-Hispanic	−4.67	(−13.24, 3.90)		
**Presence of cough**				
Yes vs no	0.16	(−2.37, 2.68)	0.01	0.90
**Inhalant group**			8.24	0.041
Conventional vs non-smoker	0.43	(−3.82, 4.67)		
E-cig vs non-smoker	−0.61	(−5.28, 4.05)		
Dual vs non-smoker	4.08	(1.12, 7.05)		

Pairwise group comparisons	Mean difference	95% CI	Raw p-value	Adjusted p-value
Dual vs nonsmoker	4.08	(1.12, 7.05)	0.007	0.042
Dual vs e-cig	4.70	(−0.39, 9.78)	0.07	0.198
Dual vs conventional	3.66	(−0.69, 8.01)	0.099	0.198
E-cig vs nonsmoker	−0.61	(−5.28, 4.06)	0.797	0.844
E-cig vs conventional	−1.04	(−6.96, 4.88)	0.731	0.844
Conventional vs nonsmoker	0.43	(−3.83, 4.68)	0.844	0.844

This table shows the multivariable linear regression model with sleep latency as the outcome, inhalant groups, age, gender, race, ethnicity, and presence of cough as the predictors. The bottom table further shows the pairwise comparisons among inhalant groups with adjusted p-values using the method of Benjamini & Hochberg. Sleep latency was determined through the following open-ended question included within the Pittsburgh Sleep Quality Index (PSQI), “During the past month, how long (in minutes) has it taken you to fall asleep each night?”.

A sensitivity analyses was conducted using responses from the three countries with the largest number of respondents (United States, United Kingdom and Canada). The results of this additional analysis resulted in the same conclusions (Supplemental Materials, Tables 1–3). As a result, the data from this study includes responses from all 47 countries.

### Dual users of e-cigarettes and conventional tobacco have greater drug use

**Figure 2:**
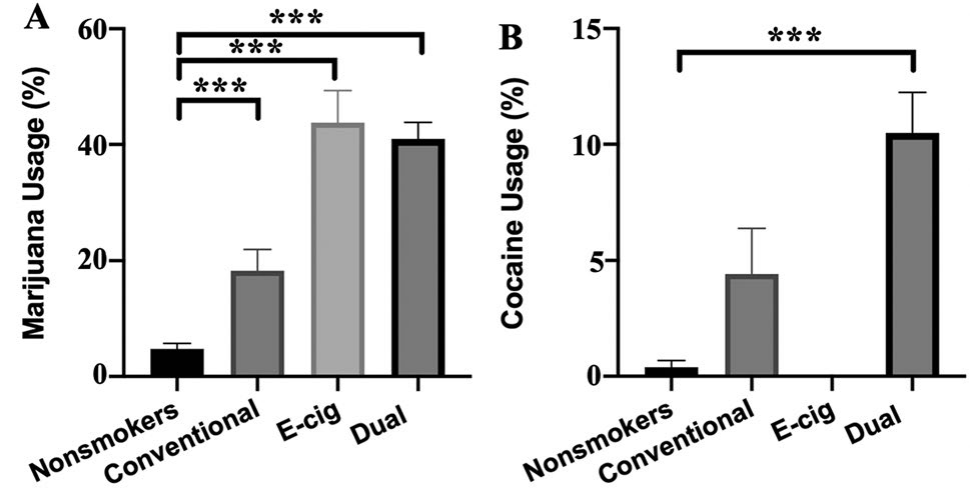
Dual users have high rates of other drug use, including marijuana. **(A)** Marijuana Usage across Inhalant groups. Dual users reported more marijuana usage when compared to non-smokers. (**B)** Cocaine usage across Inhalant groups. Dual users reported higher cocaine usage than non-smokers and e-cigarette users. *** p < 0.001.

Cross-tabulation was used to compare drug use across inhalant use groups. Regardless of gender, more dual users (42%) and e-cigarette users (45%) reported marijuana use relative to non-smokers/non-vapers (5%) and conventional tobacco users (19%; p < 0.001) (Fig. 2A). Additionally, more dual users (10%) reported use of cocaine than non-smokers/non vapers (0.4%), conventional users (5%), and e-cigarette users (0%; p < 0.001) (Fig. 2B). E-cigarette users (4%) and dual users (4%) had higher methamphetamine use than conventional tobacco smokers (2%) and non-smokers/non-vapers (0.4%; p = 0.007). More dual users (4%) reported use of *N,N*-dimethyltryptamine (DMT) than non-smokers/non vapers (0.2%), conventional tobacco smokers (1%), and e-cigarette users (1%). Two participants reported heroin/morphine/fentanyl use, and both were dual users. Seven participants reported using prescription tablet opiates, four reported using recreational opiates, and two reported using PCP, with a majority of these being dual users.

### Presence of cough is associated with poorer sleep quality and dual inhalant use

Analysis of PSQI scores and presence of cough, as determined by LCQ, revealed that participants with a cough had higher PSQI scores (7.64, SD = 3.661), indicating worsened sleep quality, compared to those without a cough (mean difference mean difference = 0.82, 95%CI: (−0.33, 1.31), p = 0.001). These data suggest that cough may have a direct adverse impact on sleep quality in this study sample. However, we did not find a significant association between the inhalant groups and PSQI scores (p = 0.313) (Table 3). There was also no evidence of interaction between inhalant groups and gender on PSQI scores (p = 0.929). When gender was assessed as a variable, males were found to have lower PSQI scores (better sleep quality) relative to females (mean difference of −0.92, 95%CI: −1.41, −0.44, p < 0.001).

**Table 3:** Linear regression model to assess the association between inhalant groups and PSQI scores or sleep quality.

	Mean difference	95% CI	Wald Chi-squared	p-value
Age	0.01	(−0.02, 0.03)	0.10	0.747
**Gender**				< 0.001
Male vs female	−0.92	(−1.41, −0.44)	13.91	
**Race**			0.70	0.705
Asian vs. White	0.01	(−0.59, 0.61)		
Other vs. White	0.32	(−0.45, 1.09)		
**Ethnicity**			2.46	0.292
Hispanic vs. non-Hispanic	−0.58	(−1.40, 0.24)		
Unknown vs. non-Hispanic	−0.71	(−2.31, 0.90)		
**Presence of cough**				
Yes vs no	0.82	(0.33, 1.31)	10.65	< 0.001
**Inhalant group**			3.56	0.313
Conventional vs non-smoker	−0.51	(−1.32, 0.30)		
E-cig vs non-smoker	0.35	(−0.56, 1.26)		
Dual vs non-smoker	0.22	(−0.36, 0.80)		

Pairwise group comparisons	Mean difference	95% CI	Raw p-value	Adjusted p-value
Dual vs nonsmoker	0.22	(−0.36, 0.80)	0.452	0.542
Dual vs E-cig	−0.13	(−1.12, 0.86)	0.797	0.797
Dual vs conventional	0.73	(−0.10, 1.56)	0.085	0.417
E-cig vs nonsmoker	0.35	(−1.32, 0.30)	0.448	0.542
E-cig vs conventional	0.86	(−0.28, 1.27)	0.139	0.417
Conventional vs nonsmoker	−0.51	(−1.32, 0.30)	0.220	0.440

This table shows the multivariable linear regression model with PSQI score as the outcome, inhalant groups, age, gender, race, ethnicity, and presence of cough as the predictors. The bottom table further shows the pairwise comparisons among inhalant groups with adjusted p-values using the method of Benjamini & Hochberg. PSQI is a tool to assess sleep quality and disturbances over a one-month interval; the self-rated questionnaire yields scores from 0 to 21 with higher scores indicating poorer sleep quality.

Inhalant use was associated with higher reporting of cough. Dual users had the highest reporting of cough in the last 30 days (48%, n = 134 of 278), with e-cigarette users having the lowest reporting of cough of all inhalant users (24%, n = 18 of 74); non-inhalant users and conventional tobacco users had similar cough reportings in the last 30 days (39%, n = 183 of 472, and 37%, n = 40 of 109, respectively). Logistic regression model adjusting for age and gender showed dual users had a higher presence of cough compared to non-smokers/non-vapers (OR = 1.47, 95% CI: 1.06 to 2.04, raw p = 0.019, adjusted p = 0.038) and e-cigarette users (OR = 3.03, 95% CI: 1.65 to 5.56, raw and adjusted p < 0.001). E-cigarette users reported a lower presence of cough than non-smokers/non-vapers (OR = 0.49, 95% CI: 0.28 to 0.86, raw p = 0.013, adjusted p = 0.038) (Table 4). There is no interaction between gender and inhalant groups on reported incidence of cough (p = 0.839).

**Table 4:** Multivariable logistic regression model to assess the association between inhalant groups and presence of cough by LCQ in the past 30 days.

	AOR	95% CI	Wald Chi-squared	p-value
Age	0.99	(0.97, 1.00)	5.50	0.019
Gender	1.29	(0.98, 1.70)	3.32	0.068
**Race**			1.18	0.55
Asian vs. White	0.83	(0.59, 1.17)		
Other vs. White	0.96	(0.62, 1.49)		
**Ethnicity**			10.09	0.006
Hispanic vs. non-Hispanic	0.55	(0.34, 0.89)		
Unknown vs. non-Hispanic	0.29	(0.09, 0.87)		
**Inhalant group**			14.60	0.002
Conventional vs non-smoker	0.96	(0.60, 1.52)		
E-cig vs non-smoker	0.49	(0.28, 0.86)		
Dual vs non-smoker	1.47	(1.07, 2.03)		

Pairwise group comparisons	OR	95% CI	Raw p-value	Adjusted p-value
Dual vs nonsmoker	1.47	(1.06, 2.04)	0.019	0.038
Dual vs E-cig	3.03	(1.65, 5.56)	< 0.001	< 0.001
Dual vs conventional	1.54	(0.96, 2.46)	0.073	0.088
E-cig vs nonsmoker	0.49	(0.28, 0.86)	0.013	0.038
E-cig vs conventional	0.51	(0.25, 1.01)	0.055	0.083
Conventional vs nonsmoker	0.96	(0.61, 1.52)	0.855	0.855

This table shows the multivariable logistic regression model with presence of cough in the past 30 days as the outcome, inhalant groups, age, gender, race, and ethnicity as the predictors. The bottom table further shows the pairwise comparisons among inhalant groups with adjusted p-values using the method of Benjamini & Hochberg. Leicester Cough Questionnaire (LCQ) is a tool to assess presence of cough and cough related quality of life; the total score range is from 3 to 21, with higher scores indicating a higher quality of life.

## Discussion

These data demonstrate that dual use of e-cigarettes with conventional tobacco is associated with increased cough as well as increased sleep latency. Historically, conventional tobacco use is known to cause both cough and increased sleep latency^34,35^. Dual users have been found to have higher levels of nicotine exposure relative to use of either inhalant alone^10^, and the increased time to fall asleep may be directly due to the activating effects of nicotine in the central nervous system (CNS). It is also possible, however, that the higher incidence of cough may alone result in the longer sleep latency found in this cohort^36^. Our study did not find any significant associations between overall sleep quality, as measured by PSQI scores, and inhalant usage.

While previous studies have highlighted the associations of sole e-cigarette^37^ or conventional tobacco^11^ use and adverse sleep outcomes, there is little research on the impact of dual usage. For example, while Brett et al. found that e-cigarette use worsened sleep quality in young adults, the study excluded dual users^38^. There are two studies that sought to address the impact of dual usage on sleep health; however, each had limitations such as they either focused on specific age groups, assessed only one type of inhalant use, or used generic questions (non-validated questionnaires) for evaluating sleep^39,40^. Moreover, the articles also concluded that further investigation was required to properly assess the impact of e-cigarette or dual usage on sleep. As a result, our study contributes to the current literature by robustly assessing sleep quality using the validated PSQI questionnaire in all age groups.

Recognizing the altered sleep patterns in adult e-cigarette users, in particular dual users, raises concerns since impaired sleep or sleep disturbance has been associated with worse outcomes across disease states. Studies have found that sleep disruption adversely impacts outcomes in cardiovascular disease, cancer, and infectious disease^41^. Furthermore, increased sleep latency in dual users is concerning as difficulty falling asleep can be indicative of autonomic dysfunction^31^.

As dual users reported the highest use of drugs such as cocaine and methamphetamine relative to all other groups, the gateway hypothesis may be a possible explanation^42^. Since dual users have been found to have higher levels of nicotine exposure^10^, it is possible that dual use may create an increased priming effect for drug use and increased facilitation of stimulant addiction compared to sole inhalant use of tobacco or e-cigarettes. Another explanation may be that dual users believe that other drugs are either less risky or more pleasurable than e-cigarettes and conventional tobacco. Further studies are needed to explain the causal mechanism of dual use of e-cigarettes and conventional tobacco leading to other drug use.

Our study has several limitations. First, we recruited our study sample through social media platforms, which can cause convenience sampling bias and limit our conclusions on the population studied. Due to social-media recruitment, we were unable to calculate an accurate response rate since a denominator, or the number of people the survey was sent to, could not be identified. Also, survey research relies on self-reporting, such that some inaccuracy is likely, but misclassification should be random and biased towards the null hypothesis. Furthermore, the low number of observations from underrepresented minorities (Alaska Native, Pacific Islander, Middle Eastern, Hispanic, African American and Mixed ethnicities) means that the results of our study may not be applicable to these groups. Since our study design was cross-sectional, it is difficult to understand the temporal relationship between inhalant use and sleep. For instance, some inhalant users might utilize e-cigarette devices or conventional tobacco products as a coping mechanism when having trouble sleeping. Moreover, while IP addresses were recorded to prevent more than one response per device, the same participant could have used multiple IP addresses to increase their chance of winning the lottery. However, each survey response was reviewed thoroughly by the research team to assess for valid entries to each question*.*

Another limitation is that our survey did not include questions regarding comorbidities; consequently, significant associations found within the study regarding sleep latency or cough may be due this confounding variable. Our survey was also designed for English-based participants and was posted on American-based social media platforms. We posit that the international respondents who accessed and took the survey are likely to be English speaking. However, we still include this as a limitation to our results since our survey has not been validated for use in different countries and across cultures. Finally, while we hypothesized that vaping would lead to higher incidence of cough, which would impact sleep quality, our study was not sufficiently powered to perform formal mediation analyses. Mechanistic studies would be required to draw any rigorous causal inferences. However, we still view cough as an important patient-reported outcome even though our data fall short of allowing us to define definitive causal pathways.

While this study has several limitations, our findings validate those of our smaller pilot study in 2018, further suggesting that dual usage affects sleep latency^9^. Dual use was also associated with increased cough, such that cough may be the primary contributor to increased sleep latency. Finally, dual use was associated with the highest use of cocaine, marijuana, DMT and opiates across inhalant users. This work suggests that dual usage of e-cigarettes with conventional tobacco is associated with adverse outcomes on sleep, including increased amounts of time to fall asleep. Further studies are needed to determine the mechanisms underlying these findings.

## Supplementary Information

Supplementary Information.
