# Supplementary material for: Dual use of e-cigarettes with conventional tobacco is associated with increased sleep latency in cross-sectional Study
Source: Sci Rep. 2022 Feb 15;12:2536. doi: 10.1038/s41598-022-06445-8 (PMC8847556; doi:10.1038/s41598-022-06445-8)
Supplement: Supplementary file 1 — Supplementary Information. [file 41598_2022_6445_MOESM1_ESM.docx]

**Supplemental Materials**

**Tables**

**Table 1. Linear Regression Model to assess the association between inhalant groups and PSQI scores or sleep quality with sensitivity analysis**

|  | Mean Difference | 95% CI | Wald Chi-squared | p-value |
| --- | --- | --- | --- | --- |
| **Age** | 0.05 | (-0.02, 0.03) | 0.20 | 0.659 |
| **Gender**  **Male vs Female** | -0.99 | (-1.50, -0.48) | 14.29 | <0.001 |
| **Race** |  |  | 2.15 | 0.341 |
| Asian vs. White | 0.15 | (-0.48, 0.79) |  |  |
| Other vs. White | 0.59 | (-0.20, 1.39) |  |  |
| **Ethnicity** |  |  | 1.80 | 0.407 |
| Hispanic vs. Non-Hispanic | -0.59 | (-1.45, 0.27) |  |  |
| Unknown vs. Non-Hispanic | -0.01 | (-1.75, 1.73) |  |  |
| **Presence of Cough** |  |  |  |  |
| Yes vs No | 0.86 | (0.34, 1.38) | 10.48 | <0.001 |
| **Inhalant Group** |  |  | 3.80 | 0.284 |
| Conventional vs Non-smoker | -0.55 | (-1.45, 0.35) |  |  |
| E-cig vs Non-smoker | 0.29 | (-0.65, 1.22) |  |  |
| Dual vs Non-smoker | 0.32 | (-0.30, 0.93) |  |  |

| Pairwise Group Comparisons | Mean Difference | 95% CI | Raw  p-value | Adjusted  p-value |
| --- | --- | --- | --- | --- |
| Dual vs Nonsmoker | 0.32 | (-0.30, 0.93) | 0.311 | 0.467 |
| Dual vs E-cig | 0.03 | (-0.10, 1.06) | 0.955 | 0.955 |
| Dual vs Conventional | 0.87 | (-0.06, 1.79) | 0.067 | 0.402 |
| E-cig vs Nonsmoker | 0.29 | (-0.65, 1.22) | 0.547 | 0.656 |
| E-cig vs Conventional | 0.84 | (-0.38, 1.22) | 0.178 | 0.460 |
| Conventional vs Nonsmoker | -0.55 | (-1.45, 0.35) | 0.230 | 0.460 |

Table 1 shows the multivariable linear regression model with PSQI score as the outcome, inhalant groups, age, gender, race, ethnicity, and presence of cough as the predictors. The bottom table further shows the pairwise comparisons among inhalant groups with adjusted p-values using the method of Benjamini & Hochberg. PSQI is a tool to assess sleep quality and disturbances over a one-month interval; the self-rated questionnaire yields scores from 0 to 21 with higher scores indicating poorer sleep quality.

**Table 2. Multivariable Linear Regression Model to assess the association between inhalant groups and sleep latency by PSQI with sensitivity analysis**

|  | Mean Difference | 95% CI | Wald Chi-squared | p-value |
| --- | --- | --- | --- | --- |
| **Age** | 0.07 | (-0.05, 0.18) | 1.26 | 0.26 |
| **Gender**  Male vs Female | -2.97 | (-5.60, -0.35) | 4.94 | 0.026 |
| **Race** |  |  | 4.23 | 0.121 |
| Asian vs. White | -3.00 | (-6.23, 0.23) |  |  |
| Other vs. White | 1.06 | (-3.03, 5.15) |  |  |
| **Ethnicity** |  |  | 4.78 | 0.092 |
| Hispanic vs. Non-Hispanic | -4.12 | (-8.61, 0.37) |  |  |
| Unknown vs. Non-Hispanic | -2.67 | (-12.02, 6.68) |  |  |
| **Presence of Cough** |  |  |  |  |
| Yes vs No | 0.30 | (-2.37, 2.96) | 0.05 | 0.83 |
| **Inhalant Group** |  |  | 11.58 | 0.01 |
| Conventional vs Non-smoker | -0.75 | (-5.48, 3.97) |  |  |
| E-cig vs Non-smoker | -1.09 | (-5.85, 3.67) |  |  |
| Dual vs Non-smoker | 4.85 | (1.72, 7.97) |  |  |

| Pairwise Group Comparisons | Mean Difference | 95% CI | Raw  p-value | Adjusted  p-value |
| --- | --- | --- | --- | --- |
| Dual vs Nonsmoker | 4.85 | (1.72, 7.97) | 0.002 | 0.012 |
| Dual vs E-cig | 4.93 | (0.69, 11.18) | 0.027 | 0.054 |
| Dual vs Conventional | 5.60 | (0.74, 10.46) | 0.024 | 0.054 |
| E-cig vs Nonsmoker | -1.09 | (-5.85, 3.67) | 0.655 | 0.906 |
| E-cig vs Conventional | -0.34 | (-6.67, 5.99) | 0.917 | 0.917 |
| Conventional vs Nonsmoker | -0.75 | (-5.48, 3.98) | 0.755 | 0.906 |

Table 2 shows the multivariable linear regression model with sleep latency as the outcome, inhalant groups, age, gender, race, ethnicity, and presence of cough as the predictors. The bottom table further shows the pairwise comparisons among inhalant groups with adjusted p-values using the method of Benjamini & Hochberg. Sleep latency was determined through the following open-ended question included within the Pittsburgh Sleep Quality Index (PSQI), *“During the past month, how long (in minutes) has it taken you to fall asleep each night?”*.

**Table 3. Multivariable Logistic Regression Model to assess the association between inhalant groups and presence of cough by LCQ in the past 30 days with sensitivity analysis**

|  | AOR | 95% CI | Wald Chi-squared | p-value |
| --- | --- | --- | --- | --- |
| **Age** | 0.98 | (0.97, 1.00) | 6.04 | 0.014 |
| **Gender** | 1.21 | (0.91, 1.62) | 1.68 | 0.196 |
| **Race** |  |  | 1.18 | 0.55 |
| Asian vs. White | 0.83 | (0.58, 1.19) |  |  |
| Other vs. White | 1.04 | (0.66, 1.65) |  |  |
| **Ethnicity** |  |  | 12.31 | 0.002 |
| Hispanic vs. Non-Hispanic | 0.52 | (0.31, 0.86) |  |  |
| Unknown vs. Non-Hispanic | 0.15 | (0.03, 0.65) |  |  |
| **Inhalant Group** |  |  | 16.37 | 0.001 |
| Conventional vs Non-smoker | 0.83 | (0.49, 1.40) |  |  |
| E-cig vs Non-smoker | 0.46 | (0.26, 0.82) |  |  |
| Dual vs Non-smoker | 1.51 | (1.07, 2.12) |  |  |

| Pairwise Group Comparisons | OR | 95% CI | Raw  p-value | Adjusted  p-value |
| --- | --- | --- | --- | --- |
| Dual vs Nonsmoker | 1.51 | (1.07, 2.12) | 0.019 | 0.038 |
| Dual vs E-cig | 3.29 | (1.75, 6.17) | <0.001 | <0.001 |
| Dual vs Conventional | 1.81 | (1.06, 3.08) | 0.029 | 0.044 |
| E-cig vs Nonsmoker | 0.46 | (0.26, 0.82) | 0.009 | 0.027 |
| E-cig vs Conventional | 0.55 | (0.26, 1.16) | 0.114 | 0.137 |
| Conventional vs Nonsmoker | 0.83 | (0.49, 1.40) | 0.491 | 0.491 |

Table 3 shows the multivariable logistic regression model with presence of cough in the past 30 days as the outcome, inhalant groups, age, gender, race, and ethnicity as the predictors. The bottom table further shows the pairwise comparisons among inhalant groups with adjusted p-values using the method of Benjamini & Hochberg. Leicester Cough Questionnaire (LCQ) is a tool to assess presence of cough and cough related quality of life; the total score range is from 3-21, with higher scores indicating a higher quality of life.

**STROBE Statement**—Checklist of items that should be included in reports of ***cross-sectional studies***

|  | Item No | Recommendation | Page No |
| --- | --- | --- | --- |
| **Title and abstract** | 1 | (*a*) Indicate the study’s design with a commonly used term in the title or the abstract | 1 |
|  |  | (*b*) Provide in the abstract an informative and balanced summary of what was done and what was found | 2 |
| Introduction | | | |
| Background/rationale | 2 | Explain the scientific background and rationale for the investigation being reported | 3 |
| Objectives | 3 | State specific objectives, including any prespecified hypotheses | 3 |
| Methods | | | |
| Study design | 4 | Present key elements of study design early in the paper | 5 |
| Setting | 5 | Describe the setting, locations, and relevant dates, including periods of recruitment, exposure, follow-up, and data collection | 5 |
| Participants | 6 | (*a*) Give the eligibility criteria, and the sources and methods of selection of participants | 5 |
| Variables | 7 | Clearly define all outcomes, exposures, predictors, potential confounders, and effect modifiers. Give diagnostic criteria, if applicable | 6-7 |
| Data sources/ measurement | 8* | For each variable of interest, give sources of data and details of methods of assessment (measurement). Describe comparability of assessment methods if there is more than one group | 6 |
| Bias | 9 | Describe any efforts to address potential sources of bias | 5 |
| Study size | 10 | Explain how the study size was arrived at | 7-8 |
| Quantitative variables | 11 | Explain how quantitative variables were handled in the analyses. If applicable, describe which groupings were chosen and why | 7-8 |
| Statistical methods | 12 | (*a*) Describe all statistical methods, including those used to control for confounding | 8 |
|  |  | (*b*) Describe any methods used to examine subgroups and interactions | 8 |
|  |  | (*c*) Explain how missing data were addressed |  |
|  |  | (*d*) If applicable, describe analytical methods taking account of sampling strategy | 7-8 |
|  |  | (*e*) Describe any sensitivity analyses | 8 |
| Results | | | |
| Participants | 13* | (a) Report numbers of individuals at each stage of study—eg numbers potentially eligible, examined for eligibility, confirmed eligible, included in the study, completing follow-up, and analysed | 8 |
|  |  | (b) Give reasons for non-participation at each stage | 8 |
|  |  | (c) Consider use of a flow diagram | X |
| Descriptive data | 14* | (a) Give characteristics of study participants (eg demographic, clinical, social) and information on exposures and potential confounders | 8-9 |
|  |  | (b) Indicate number of participants with missing data for each variable of interest | 9 |
| Outcome data | 15* | Report numbers of outcome events or summary measures | 9 |
| Main results | 16 | (*a*) Give unadjusted estimates and, if applicable, confounder-adjusted estimates and their precision (eg, 95% confidence interval). Make clear which confounders were adjusted for and why they were included | 9-11 |
|  |  | (*b*) Report category boundaries when continuous variables were categorized | X |
|  |  | (*c*) If relevant, consider translating estimates of relative risk into absolute risk for a meaningful time period | X |
| Other analyses | 17 | Report other analyses done—eg analyses of subgroups and interactions, and sensitivity analyses | 11 |
| Discussion | | | |
| Key results | 18 | Summarise key results with reference to study objectives | 11 |
| Limitations | 19 | Discuss limitations of the study, taking into account sources of potential bias or imprecision. Discuss both direction and magnitude of any potential bias | 12-13 |
| Interpretation | 20 | Give a cautious overall interpretation of results considering objectives, limitations, multiplicity of analyses, results from similar studies, and other relevant evidence | 11-12 |
| Generalisability | 21 | Discuss the generalisability (external validity) of the study results | 12-13 |
| Other information | | | |
| Funding | 22 | Give the source of funding and the role of the funders for the present study and, if applicable, for the original study on which the present article is based | 1 |

*Give information separately for exposed and unexposed groups.

**Note:** An Explanation and Elaboration article discusses each checklist item and gives methodological background and published examples of transparent reporting. The STROBE checklist is best used in conjunction with this article (freely available on the Web sites of PLoS Medicine at http://www.plosmedicine.org/, Annals of Internal Medicine at http://www.annals.org/, and Epidemiology at http://www.epidem.com/). Information on the STROBE Initiative is available at www.strobe-statement.org.

**Appendix A**

Survey Questionnaire

Participants recruited to enter the study were asked to complete the following questionnaire, included below. The purpose of the survey was to assess potential differences amongst inhalant use, sleep quality, and cough symptoms through the UCSD Inhalant Questionnaire, Pittsburgh Sleep Quality Index, and Leighton Cough Questionnaire, respectively.

**UCSD Inhalant Survey**

1. Thank you for your interest in this sleep survey. Completion of this survey will enter you

into a raffle for a $250 Amazon gift card.

Please review the informed consent document linked below, then click "I agree to

participate" to start the UCSD IRB approved survey.

As described in the Informed Consent, all research data from this one-time online survey is

anonymous and will be kept confidential. Click "I agree to participate" to start the survey.

- I agree to participate.
- I'm not interested in participating in this survey (this will end the survey).

2. How old are you? (Enter a number, in years)

- ________

3. What is your gender?

- Male
- Female
- Non-binary
- Prefer to self-describe ________

4. What is your race? Choose all that apply.

- American Indian or Alaska Native
- Asian Indian
- Asian
- Black or African American
- Native Hawaiian or Pacific Islander
- White or Caucasian
- Prefer not to answer
- Prefer to self-describe ________

5. Are you Hispanic or of Spanish origin? Choose all that apply.

- No, not of Hispanic or Spanish origin
- Yes - Mexican, Mexican American
- Yes - Puerto Rican
- Yes - Cuban
- Yes - another Hispanic or Spanish origin
- Don’t know

6. Please check the corresponding boxes if you have asthma, COPD, or other lung diseases (if other, please list the diagnosis).

- No Lung Disease
- Yes - Asthma
- Yes - COPD, Emphysema
- Yes - Other (please specify) ________

7. Have you ever used any form of tobacco?

Including but not limited to: cigarettes, cigars, pipes, hookah and shisha?

- Yes
- No *[Skips to Question 25]*

8. Are you actively using any form of tobacco?

(>1 use per month in the last year)

Including, but not limited to: cigarettes, cigars, pipes, hookah, shisha.

- Yes *[Skips to Question 18]*
- No

9. Did you Smoke cigarettes?

- Yes *[Avoids Questions 18-24 and skips to 25 after 10-13 or 10-17 (if yes on 13)]*
- No *[Skips to Question 14, then avoids Questions 18-24 and skips to 25 after 14-17]*

10. How many cigarettes did you smoke per day? <1

- 1-10
- 11-20
- 21-40
- >40
- Other (please specify) ________

11. What year did you START smoking cigarettes?

- ________

12. What year did you STOP smoking cigarettes?

- ________

13. Did you smoke cigars/ cigarillos, a pipe, hookah or any other forms of tobacco?

- Yes
- No *[Skips to 25]*

14. What types of tobacco did you use?

- Cigars/ Cigarillos
- Pipe
- Hookah/ Shisha
- Other (please specify) ________

15. What year did you START smoking these forms of tobacco?

- ________

16. What year did you STOP smoking these forms of tobacco?

- ________

17. How many times per day did you smoke?

- <1
- 1-2
- 2-3
- 4-5
- >5
- Other (please specify) ________

18. Do you Smoke cigarettes?

- Yes
- No *[Skips to Question 22]*

19. How many cigarettes per day do you smoke?

- <1
- 1-10
- 11-20
- 21-40
- >40
- Other (please specify) ________

20. What year did you start smoking cigarettes?

- ________

21. Do you smoke cigars/ cigarillos, a pipe, hookah or any other forms of tobacco?

- Yes
- No *[Skips to Question 25]*

22. What types of tobacco do you use?

- Cigars/ Cigarillos
- Pipe
- Hookah/ Shisha
- Other (please specify) _______

23. How many times per day do you smoke?

- <1
- 1-2
- 2-3
- 4-5
- >5
- Other (please specify) ________

24. What year did you start smoking cigarettes?

- ________

25. Have you ever used an e-cigarette? (Including, but not limited to: JUUL, vape pen, e-hookah, e-pipe, e-cigs, etc.)

- Yes
- No *[Skips to Question 41]*

26. Are you actively using any e-devices? (>1 use per month in the last year) (Including but not limited to: JUUL, vape-pen, e-hookah, e-pipe, etc.)

- Yes *[Skips to Question 34]*
- No *[Avoids Questions 34-40 and skips to 41 after 27-33]*

27. What type(s) of e-device(s) did you typically use?

- E-cigarette
- Vape pen
- JUUL
- Mod
- Suorin / Drop
- Smok / Nord
- Other (please specify) ________

28. What percentage of nicotine did you typically use in your e-device?

- 0%
- 1-6%
- 7-12%
- >13%
- Do not know
- Other (please specify) ________

29. Did you add anything to your e-liquid?

- No
- Yes - please specify ________

30. What flavor(s) of e-liquid did you most often use?

- ________

31. How many days per week did you use your device?

- Everyday
- Less than once per week
- Some days (1-3 days per week)
- Most days (4+ days per week)
- Less than once per month
- Other (please specify) ________

32. What time did you typically start vaping?

- ________

33. What time did you typically stop vaping?

- ________

34. What type(s) of e-device(s) do you typically use?

- E-cigarette
- Vape pen
- JUUL
- Mod
- Suorin / Drop
- Smok / Nord
- Other (please specify) ________

35. What percentage of nicotine do you typically use in you e-device?

- 0%
- 1-6%
- 7-12%
- >12%
- Do not know
- Other (please specify) ________

36. Do you add anything to your e-liquid?

- No
- Yes - please specify _________

37. What flavor of e-liquid do you most use?

- ________

38. How many days per week do you use your device?

- Everyday
- Less than once per week
- Some days (1-3 days per week)
- Most days (4+ days per week)
- Less than once per month
- Other (please specify) ________

39. What time do you typically start vaping?

- ________

40. What time do you typically stop vaping?

- ________

41. Do you use marijuana? (Inhaled versions only: smoke or vape) Use is defined as >1 use per month for the last year

- Yes
- No *[Skips to Question 44]*

42. How do you inhale your marijuana / THC?

- Smoke
- Vape
- Concentrated cannibas oil (also referred to as dab, wax, shatter, resin, etc)
- Other (please specify) ________

43. How often do you smoke or vape marijuana?

- <1 day per week
- 1-2 days per week
- 3-4 days per week
- >4 days per week
- Other (please specify) ________

44. Have you used any other recreational substances in the last 2 months?

- None
- Cocaine
- Methamphetamine
- Heroin/Morphine/Fentanyl
- Oxycodone/Vicodin/Dilaudid/Percocet/Norco, etc (tablet opiates) - Prescribed for  Medical Condition
- Oxycodone/Vicodin/Dilaudid/Percocet/Norco, etc (tablet opiates) - Not prescribed
- PCP
- Volatile inhaled gases (“huffing glue”)
- Other (please specify) ________

**Pittsburg Sleep Quality Index (PSQI)**

45. The following series of questions relate to your usual sleep habits during the past month only. Your answers should indicate the most accurate reply for the majority of the days in the past month. During the past month, when have you usually gone to bed?

- 6 PM
- 7 PM
- 8 PM
- 9 PM
- 10 PM
- 11 PM
- midnight
- 1 AM
- 2 AM
- 3 AM
- 4 AM
- 5 AM
- 6 AM
- 7 AM
- 8 AM
- 9 AM
- 10 AM
- 11 AM
- noon
- Other (please specify)

46. During the past month, how long (in minutes) has it taken you to fall asleep each night?

- ________

47. During the past month, what time have you usually gotten up in the morning?

- 3 AM
- 4 AM
- 5 AM
- 6 AM
- 7 AM
- 8 AM
- 9 AM
- 10 AM
- 11 AM
- noon
- 1 PM
- 2 PM
- 3 PM
- 4 PM
- Other (please specify)

48. During the past month, how many hours were you in bed (per night)? Include time spent in bed awake (trying to fall asleep, reading, etc).

- ___________

49. During the past month, how many hours of actual sleep did you get per night? This does not include time spent in bed while awake.

- ___________

50. During the past month, how often have you had trouble sleeping because you cannot get to sleep within 30 minutes?

- Three or more times a week
- Once or twice a week
- Less than once a week
- Not during the past month

51. During the past month, how often have you had trouble sleeping because you woke up in the middle of the night or early morning?

- Three or more times a week
- Once or twice a week
- Less than once a week
- Not during the past month

52. During the past month, how often have you had trouble sleeping because you have to get up to use the bathroom?

- Three or more times a week
- Once or twice a week
- Less than once a week
- Not during the past month

53. During the past month, how often have you had trouble sleeping because you cannot breathe comfortably?

- Three or more times a week
- Once or twice a week
- Less than once a week
- Not during the past month

54. During the past month, how often have you had trouble sleeping because you feel too cold?

- Three or more times a week
- Once or twice a week
- Less than once a week
- Not during the past month

55. During the past month, how often have you had trouble sleeping because you feel too hot?

- Three or more times a week
- Once or twice a week
- Less than once a week
- Not during the past month

56. During the past month, how often have you had sleep problems due to a cough?

- Three or more times a week
- Once or twice a week
- Less than once a week
- Not during the past month

57. During the past month, how often have you had trouble sleeping because you have bad dreams?

- Three or more times a week
- Once or twice a week
- Less than once a week
- Not during the past month

58. During the past month, how often have you had trouble sleeping because you have pain?

- Three or more times a week
- Once or twice a week
- Less than once a week
- Not during the past month

59. During the past month, how often have you taken medicine (prescribed or "over-the-counter") to help you sleep?

- Three or more times a week
- Once or twice a week
- Less than once a week
- Not during the past month

60. During the past month, how often have you had trouble staying awake while driving, eating meals, or engaging in social activity?

- Three or more times a week
- Once or twice a week
- Less than once a week
- Not during the past month
- Other (please specify)

61. During the past month, how often did you have a problem keeping up enthusiasm to get things done?

- Three or more times a week
- Once or twice a week
- Less than once a week
- Not during the past month

62. During the past month, have you had disturbances or changes to your typical sleep pattern from any of the causes listed below?

- No, my sleep this month has been typical for me
- Yes, my sleep has been affected by changing my usage of tobacco, alcohol, or other drugs
- Yes, my sleep has been affected by stress at work (or school)
- Yes, my sleep has been affected by stress at home
- Yes, my sleep has been affected by illness (either my own or a family members illness)
- My sleep has been affected by other circumstances (please describe in text box below)

63. During the past month, how would you rate your sleep quality overall?

- Very
- Bad
- Fairly Bad
- Fairly Good
- Very Good

**Leicester Cough Questionnaire (LCQ)**

64. The final series of questions ask about symptoms relating to a cough. Do you recall coughing in the last 30 days?

- Yes
- No (*skip this section*)

65. In the last 2 weeks, have you had chest or stomach pains as a result of your cough?

- All of the time
- Most of the time
- A good bit of the time
- Some of the time
- A little of the time
- Hardly any of the time
- None

66. In the last 2 weeks, have you been bothered by sputum (phlegm) production when you cough?

- All of the time
- Most of the time
- A good bit of the time
- Some of the time
- A little of the time
- Hardly any of the time
- None of the time

67. In the last 2 weeks, have you been tired because of your cough?

- All of the time
- Most of the time
- A good bit of the time
- Some of the time
- A little of the time
- Hardly any of the time
- None of the time

68. In the last 2 weeks, have you felt in control of your cough?

- All of the time
- Most of the time
- A good bit of the time
- Some of the time
- A little of the time
- Hardly any of the time
- None of the time

69. How often during the last 2 weeks have you felt embarrassed by your coughing?

- All of the time
- Most of the time
- A good bit of the time
- Some of the time
- A little of the time Hardly any of the time None of the time

70. In the last 2 weeks, my cough has made me feel anxious.

- All of the time
- Most of the time
- A good bit of the time
- Some of the time
- A little of the time
- Hardly any of the time
- None of the time

71. In the last 2 weeks, my cough has interfered with my job, or other daily tasks.

- All of the time
- Most of the time
- A good bit of the time
- Some of the time
- A little of the time
- Hardly any of the time
- None of the time

72. In the last 2 weeks, I felt that my cough interfered with the overall enjoyment of my life.

- All of the time
- Most of the time
- A good bit of the time
- Some of the time
- A little of the time
- Hardly any of the time
- None of the time

73. In the last 2 weeks, exposure to paints or fumes (not smoking/e-cigs/ other drugs) has made me cough.

- All of the time
- Most of the time
- A good bit of the time
- Some of the time
- A little of the time
- Hardly any of the time
- None of the time

74. In the last 2 weeks, has your cough disturbed your sleep?

- All of the time
- Most of the time
- A good bit of the time
- Some of the time
- A little of the time
- Hardly any of the time
- None of the time
- Other (please specify)

75. In the last 2 weeks, how many times a day have you had coughing bouts?

- All of the time (continuously)
- Most times during the day
- Several times during the day
- Some times during the day
- Occasionally through the day
- Rarely
- None

76. In the last 2 weeks, my cough has made me feel frustrated.

- All of the time
- Most of the time
- A good bit of the time
- Some of the time
- A little of the time
- Hardly any of the time
- None of the time

77. In the last 2 weeks, my cough has made me feel fed up.

- All of the time
- Most of the time
- A good bit of the time
- Some of the time
- A little of the time
- Hardly any of the time
- None of the time

78. In the last 2 weeks, have you suffered from a hoarse voice as a result of your cough?

- All of the time
- Most of the time
- A good bit of the time
- Some of the time
- A little of the time
- Hardly any of the time
- None of the time

79. In the last 2 weeks, have you had a lot of energy?

- All of the time
- Most of the time
- A good bit of the time
- Some of the time
- A little of the time
- Hardly any of the time
- None of the time

80. In the last 2 weeks, have you worried that your cough may indicate serious illness?

- All of the time
- Most of the time
- A good bit of the time
- Some of the time
- A little of the time
- Hardly any of the time
- None of the time

81. In the last 2 weeks, have you been concerned that other people think something is wrong with you because of your cough?

- All of the time
- Most of the time
- A good bit of the time
- Some of the time
- A little of the time
- Hardly any of the time
- None of the time

82. In the last 2 weeks, my cough has interrupted conversation or telephone calls.

- Every time
- Most of the time
- A good bit of the time
- Some of the time
- A little of the time
- Hardly any of the time
- None of the time

83. In the last 2 weeks, I feel that my cough has annoyed my partner, family, or friends.

- Every time I cough
- Most times when I cough
- Several times when I cough
- Sometimes when I cough
- Occasionally when I cough
- Rarely
- None of the time

84. THE END.

Thank you for completing the survey. If you had problems with the survey or have questions/comments, leave them below or email inadvani@ucsd.edu If you would like to be entered into a drawing for a $250 Amazon gift card, please enter your email address below. This will not be used for any other purpose or distributed. The winner will receive a notification after the drawing is completed.

- Email address: _____________

Comments/questions (include email address if reply desired): ____________

85. Are you willing to be contacted about other UCSD study opportunities?

- If yes, please enter the email address:_____________
